# Reconstruction of Critical Nerve Defects Using Allogenic Nerve Tissue: A Review of Current Approaches

**DOI:** 10.3390/ijms22073515

**Published:** 2021-03-29

**Authors:** Tim Kornfeld, Anton Borger, Christine Radtke

**Affiliations:** Department of Plastic, Reconstructive and Aesthetic Surgery, Medical University of Vienna, Währinger Gürtel 18-20, 1090 Vienna, Austria; Tim.Kornfeld@web.de (T.K.); anton.borger@meduniwien.ac.at (A.B.)

**Keywords:** FDA, artificial nerve grafts, allograft

## Abstract

Regardless of the nerve defect length, nerve injury is a debilitating condition for the affected patient that results in loss of sensory and motor function. These functional impairments can have a profound impact on the patient’s quality of life. Surgical approaches for the treatment of short segment nerve defects are well-established. Autologous nerve transplantation, considered the gold standard, and the use of artificial nerve grafts are safe and successful procedures for short segment nerve defect reconstruction. Long segment nerve defects which extend 3.0 cm or more are more problematic for repair. Methods for reconstruction of long defects are limited. Artificial nerve grafts often fail to regenerate and autologous nerve grafts are limited in length and number. Cadaveric processed/unprocessed nerve allografts are a promising alternative in nerve surgery. This review gives a systematic overview on pre-clinical and clinical approaches in nerve allograft transplantation.

## 1. Introduction

Loss of motoric function and sensitivity in the distributed region of a peripheral nerve has a considerable impact for patients suffering peripheral nerve injuries [1]. Injuries of peripheral nerves are often accompanied with psychological stress or depression [2]. Two to five per cent of affected patients suffer a complex regional pain syndrome [3]. In 2.8% of all cases, peripheral nerve injuries are associated with severe trauma [4]. Other common reasons include infiltration by tumor, or an iatrogenic nerve lesion during medical intervention [5,6].

Despite decades of research, methods to approach and treat peripheral nerve defects are still limited [7]. In cases where the proximal and distal nerve stumps are tension free adaptable, primary nerve suturing can be performed [8]. If this is not achievable other surgical techniques must be employed. The current clinical gold standard for peripheral nerve defects with a significant gap between the proximal and distal nerve stump is autologous nerve transplantation [9,10]. A donor nerve, e.g., sural nerve, is excised, turned and sutured into the existing defect. The major disadvantage of this technique is the limitation of suitable donor nerves and donor site morbidity.

If a peripheral nerve reconstruction—via an autologous nerve transplantation or a direct end-to-end suture is not achievable, commercial artificial nerve grafts are available as a surgical alternative. The most recently marketed artificial nerve grafts were carefully reviewed by the authors in 2018 [11]. All marketed and FDA approved nerve grafts have in common that the maximal gap length that can be sufficiently reconstructed is limited to 3.0 cm in length [11].

Thus, there is the necessity to identify new materials and techniques for the reconstruction of critical nerve defects extending 3.0 cm in length. The application of cadaveric nerve allograft is a promising approach in the field of peripheral nerve surgery [12]. Decades of allograft development resulted in the first marketed commercial allograft (Axogen Avance^®^, AxoGen, Alachua, FL, USA) [13].

One major concern regarding allograft transplantation is the immune tolerance in the host. An acute rejection of allogenic tissue is mainly mediated by a humoral and cellular response of the host [14,15]. Schwann cells express major histocompatibility complex II (MHC II) on their outer surface mediating the immunogenic response leading to an allograft rejection [15]. To avoid immunogenic rejection different procedures were developed over the last decades.

The use of calcineurin inhibitors like tacrolimus (FK506) in allograft-based nerve reconstruction was temporary popular. Tacrolimus is a customized immunosuppressant to avoid tissue rejection after allogenic transplantation [16]. The major side effects include opportunistic infections in the host [17]. That might be the main reason why other established procedures to deplete immunogenic components of allogenic tissue in the host such as decellularization methods by cold preservation, freeze-thawing and detergent prevailed.

The use of cadaveric decellularized allografts might be a suitable approach for nerve defect reconstruction of critical nerve defects because the major challenge in reconstructive surgery is still the handling of critical and multiple nerve lesions. Previous and current approaches are reviewed here in order to provide guidelines for clinical translation.

## 2. Methods for Preparation and Transplantation of Nerve Allografts

### 2.1. Cold Preservation

The gold standard for allograft preservation for more than 40 years is the cold preservation of solid organs, or human tissue for transplant [18,19]. Human tissue is stored in preservation medium at 0–4 °C. The basic principle behind this procedure is a down regulation of metabolic processes within the tissue and the maintained supply of the intracellular environment resulting in a prolonged graft survival time [20]. This downregulation is significantly influenced by the storage medium. Collins and colleagues [21] first introduced the Collins solution in 1969 as a cold storage solution mainly consisting of potassium phosphate with a high magnesium and low sodium concentration. The Collins solution was later improved to the Euro-Collins cold preservation solution (EC-solution). The Euro-Collins solution is characterized by an increased glucose concentration and an absence of magnesium. The EC-solution was able to increase the allograft survival time to 30–96 h in a canine kidney model [22].

More recently, the EC-solution was replaced by the University of Wisconsin solution (UW-solution) [23]. The UW-solution consists mainly out of potassium lactobiante and potassium phosphate. The UW-solution was for a long period the gold standard in cold preservation of human organs for transplantation [24]. In direct comparison to the EC-solution, Toom et al. demonstrated better graft survival with regard to the metabolic activity in hepatic cells by cold preservation in UW-solution [25].

In 1999, Atchahabian et al. [26] found out that a prolonged period of cold preservation in UW-solution lead to a significant down regulation of MHC II in rodent nerve allografts in addition to the previously described preservation of the intracellular environment. In 2007, Ikegutchi et al. [27] refined the UW-solution by adding polyphenol for prolonged cold preservation of nerve allografts. Four weeks cold-preserved nerve allografts were then transplanted in 2.0 cm sciatic nerve defect in Lewis rats and 24 weeks following surgery a degree of nerve regeneration comparable to the isograft control was seen [27].

### 2.2. Freeze-Thawing

Freezing and freeze-thawing was introduced as a subsequent pretreatment method for peripheral nerve allografts between 1970–1985 [28,29,30]. The major aim is to achieve a reduction of immunogenicity in the host [31].

### 2.3. Detergent Based Decellularization

Most recent methods for allograft processing are detergent based decellularization procedures. Initial approaches mostly based on Triton X-100 protocols which evolved over the last several years [32]. The Axogen Avance^®^ nerve graft as the first FDA approved processed nerve allograft undergoes detergent based decellularization by Triton X and sulfobetaine-16 and sulfobetaine-10 following the protocol established by Hudson et al. [33]. In a subsequent step, chondroitin sulfate proteoglycan (CSPG) is removed by an enzymatic digestion according to the protocol of Neubauer et al. [34]. Finally, the Avance nerve graft undergoes gamma-irradiation for sterilization [34,35,36].

## 3. Results

### 3.1. Systematic Search

**Figure 1:**
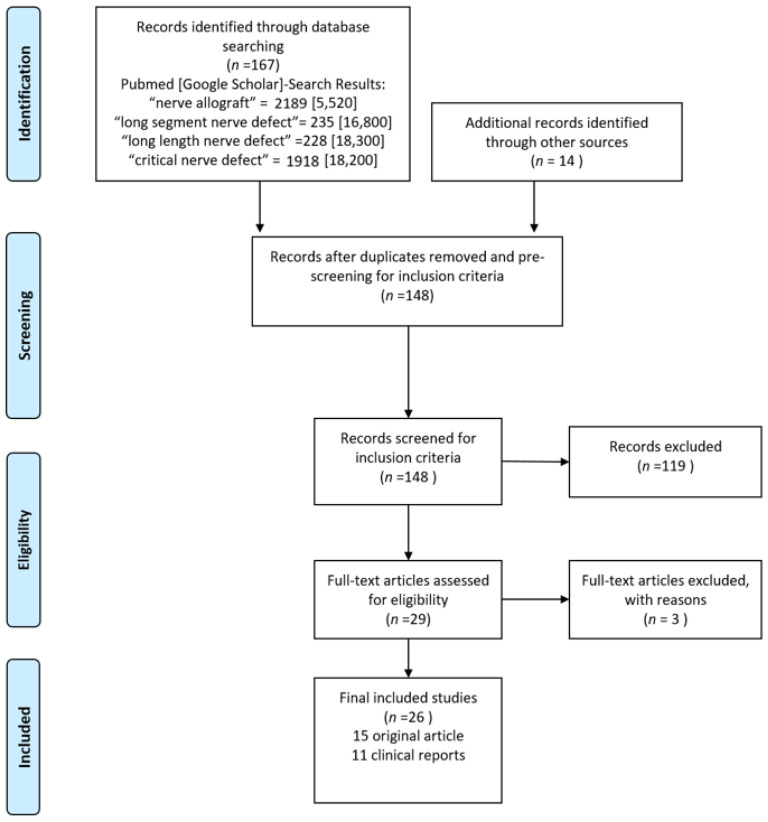
PRISMA flow chart for peripheral nerve defects extending 4.0 cm in length and reconstruction by cadaveric processed/unprocessed allografts accordingly to the PRIMSA statements described by Moher et al. [37].

In a systematic search 148 records were identified. Twenty-nine articles met the inclusion criteria of long length allograft reconstruction (>4.0 cm) pre-clinical (Table 1) or clinical (Table 2) settings. Three full texts were excluded through identification as duplicates or incomparable methods. In total 26 articles were included: 15 original article and 11 case reports/clinical studies. The systematic search process is presented in a PRISMA flow chart accordingly to the PRISMA-statements of Moher et al. [37] (Figure 1).

### 3.2. Nerve Allotransplantation on Critical Nerve Defects in a Pre-Clinical Setting

In 1982, Zalewski et al. [30] investigated the use and the outcome of fresh and frozen allografts (−70 °C for 3 days) in the rodent animal. The tibial nerve of Fisher rats was reconstructed using a 4.0 cm fresh or frozen allograft. Lewis rats served as allogenic donor. An autograft of the same length served as control. After 2 and 9 months, the animals were sacrificed and the explanted nerve grafts were analyzed. Zalewski et al. was able to demonstrate that nerve regeneration failed using fresh and frozen allografts [30] and nerve regeneration was only observed in the isograft control group. On the basis of this findings, Zalewski et al. [30] concluded that the use of allografts is not recommended for a clinical setting.

As a subsequent study, the same authors investigated nerve regeneration of a 4.0 cm peroneal nerve defect in the immunotolerant Lewis rat using a fresh allograft [38]. An isograft reconstruction and an allograft reconstruction in the normal rodent animal served as control. The results indicated a sufficient nerve regeneration in the immunotolerant animals and the isograft control group. Allografts were rejected in immunocompetent control group. Based on their results Zalewski et al. concluded that the use of a nerve allograft is only successful in the immunosuppressed host [38].

Freeze-thawed acellular nerve allografts were used for reconstruction of a 2.0 cm nerve defect in rats and in a 4.0 cm nerve defect in rabbits by Gulati et al. [39]. Nerve autograft and non-processed allograft were used as control. Nerve regeneration was controlled after 2, 4, 12 and 20 weeks. All cellular allografts were rejected. Significant nerve regeneration was observed in the autograft group and the acellular allograft group, respectively. Following the discussion of Gulati et al., acellular nerve allografts show significant nerve regeneration with a significant higher axonal number compared to the other groups. This is explained by a reduced immunogenic activity in the host organism [39].

In 1996, Strasberg et al. [40] performed an 8.0 cm median nerve reconstruction in the sheep. Animals were divided in four subgroups and a median nerve defect was either reconstructed with an untreated allograft, autograft, a cold-preserved allograft or a cold preserved autograft. Cold preservation of nerve grafts was performed at 5 °C for 7 days in University of Wisconsin solution. The nerve regeneration was controlled 5- and 10-months following surgery. The autograft demonstrated sufficient nerve regeneration over a distance of 8.0 cm. In the untreated allograft group and in the cold preserved allograft group, axonal regeneration was absent. Cold preservation did not enhance the axonal elongation. Strasberg et al. concluded that an immunosuppression might enhance the nerve regeneration in the cold preserved allograft group.

Just two years later, Atchabahian et al. [41] investigated the use of an 8.0 cm fresh nerve allograft in a ulnar nerve defect in a porcine model without preservation or immunosuppression. An 8.0 cm ulnar nerve autograft served as control. Regeneration was examined 6- and 10-months following surgery. The results demonstrated poor regeneration in the allograft group while the control group showed sufficient nerve regeneration in all specimens. Based on the results, they described successfully the reconstruction of an 8.0 cm critical nerve defect by an autologous nerve transplantation in a porcine model.

In the same year, Ide et al. [42] performed an experimental investigation in the canine animal model. Here, a 5.0 cm peroneal nerve defect was either reconstructed using an isograft of the same length or a freeze-thawed nerve allograft. Allografts were freeze-thawed three times and stored for 3 months at −20 °C before transplantation. Nerves were then transplanted in a 5.0 cm peroneal nerve defect. Allografts were transplanted either by adding a low dose, or high dose of basic fibroblast growth factor (bFGF) or without any co-factor. Nerve regeneration was examined after 1 and 3 months by determination of axon counting and immunostaining for neurofilament. The one-month group demonstrated poor regeneration in all allograft groups, only the autograft group demonstrated axonal sprouting. In the three-month group, sufficient nerve regeneration could be observed in all groups (autograft 22.6 ± 8.9; allograft w/o 10.6 ± 3.8, low dose bFGF 10.4 ± 6.7; high dose bFGF 19.2 ± 9.3). An important point is that from all groups, the autograft group showed superior regeneration. With this experimental setting, Ide et al. [42] was able to demonstrate sufficient nerve regeneration using allografts in a critical nerve defect without the need of immunosuppression. Further, the addition bFGF lead to enhanced nerve regeneration in the canine animal model.

In the early days of systemic immunosuppression, Matsuyama et al. [43] investigated the use of fresh allograft in combination with cyclosporine A in a 5.0 cm ovine nerve defect model. The 5.0 cm nerve defects were either reconstructed using an 8.0 cm unprocessed allograft from the same stock or an 8.0 cm fresh autograft. Allograft rejection was prevented by maintaining a blood level of 1000 ng/L of cyclosporine A. Due to extensive opportunistic infections, the experiment was truncated prior to the planned endpoint of 6 month. Allografts without immunosuppression were entirely rejected. The best nerve regeneration was achieved in the autograft + cyclosporine A group. Nevertheless, the level of immunosuppression has to be reconsidered.

Brenner et al., 2005 [44] introduced the porcine animal model in the research of peripheral nerve regeneration. They investigated the reconstruction of a 5.0 cm ulnar nerve defect using a cold preserved nerve allograft in combination with MHC-matched Schwann cells with or without a preoperative injection of an ultraviolet-B irradiated donor alloantigen. All nerve allografts were cold preserved in University of Wisconsin solution for two weeks at 4 °C. Nerve regeneration was observed for 20 weeks. The results indicate a robust nerve regeneration in all cold preserved allografts. Allografts in combination with an MHC-matched Schwann cell injection demonstrated a significant higher number of regenerated nerve fibers. The preoperative injection of ultraviolet-B irradiated donor alloantigen was not able to enhance peripheral nerve regeneration. Despite the negative results of ultraviolet-B irradiated donor alloantigen injection, Brenner et al., 2005 [44] was able to introduce the miniature pig as a translational research model in peripheral nerve surgery and demonstrated successful nerve allograft transplantation without rejection and immunosuppression.

Based on the results of Matsuyama et al. [43], Jensen et al. [45] investigated nerve regeneration on an 8.0 cm ulnar nerve defect in the porcine animal model with an additional immunosuppression using tacrolimus (0.1 to 0.4 mg/kg). All animals received an 8.0 cm fresh, unprocessed ulnar nerve allograft or autograft. Animals without immunosuppression served as control. Nerve grafts were harvested 24 weeks following surgery. The results demonstrated enhanced nerve regeneration in the tacrolimus group surpassing the outcome of untreated animals. Especially the autograft-tacrolimus group demonstrated superior regeneration resulting in a doubled axonal fiber count and nerve density compared to the controls. All allografts without immunosuppression were rejected. By this study, Jensen et al. [45] was able to demonstrate that a moderate immunosuppression using tacrolimus is able to enable nerve regeneration using a fresh, unprocessed allograft and to enhance nerve regeneration in isografts.

On the basis of the investigation of Brenner et al., 2005 [44], Tung et al. performed a subsequent experiment [46]. Comparable to the previous study, they investigated nerve regeneration after an intravenous pretreatment with ultraviolet B irradiated donor alloantigens in a porcine model. A 6.0 cm fresh untreated median nerve allograft was used in this experimental setting. Ten months following surgery, control group demonstrated a failed axonal regeneration and a rejection of the fresh unprocessed allograft. The experimental group, previously treated with ultraviolet B irradiated donor alloantigens, showed successful axonal regeneration throughout the 6.0 cm nerve gap. Nevertheless, the axonal elongation was still described inferior to the current clinical gold standard. However, Tung et al. established with this study as a new concept of immunosuppression in peripheral nerve surgery [46].

In 2006, Auba et al. [47] were the first group to translate the experimental use of nerve allografts to a nonhuman primate model. In this study, 8 non-human primates received a 4.0 cm ulnar nerve allograft. Nerve regeneration was evaluated after 3, 5, and 8 months and regeneration was determined by electrophysiology and axon counting. Eight months following surgery, nerve allografts were harvested and histologically analyzed. Allografts used in this study were cold preserved in University of Wisconsin solution at 4 °C for three weeks. All animals that received a cold preserved allograft were immunosuppressed using a concentration of 9 mg/kg/day tacrolimus for at least 2 months. The results demonstrated a similar axonal count of the allograft group compared to the isograft control group. Regarding the electrophysiological analysis, the autograft group demonstrated superior regeneration measured by nerve conductive velocity (NCV). Auba et al. [47] considered that a partial rejection of the allograft in some specimens might be the result of an early truncation of the immunosuppression following two months of surgery.

Hess et al. [48] continued experimental studies in the non-human primate model. In this study, a 6.0 cm ulnar nerve defect was either reconstructed with an autograft or a same-sized fresh unprocessed allograft, cold-preserved allograft, or a cold-preserved allograft seeded with autologous Schwann cells. Cold preserved nerve allografts were stored in University of Wisconsin solution at 4 °C for 7 weeks. Nerve regeneration was controlled 6 months following surgery. The results indicate that cold-preserved allografts seeded with autologous Schwann cells were able to mediate a sufficient axonal regeneration within the time of observation. Hess et al. [48] revealed that a prolonged preservation time of 7 weeks might be beneficial regarding regenerative capacities in the allograft. Despite this achievement in preprocessing, the autograft (fiber count: 8059 ± 5557) still demonstrated significantly better results compared to the cold preserved allograft (fiber count: 1488 ± 2549) and the cold preserved allograft + autologous Schwann cells (fiber count: 3525 ± 2352). Different as expected, unprocessed nerve allografts demonstrated comparable results to the autograft group (fiber count: 6115 ± 3611). Following the discussion of Hess et al. [48], this is mainly explained by an unexpected consanguinity between specimens. Finally, Hess et al. [48] was able to demonstrate sufficient regeneration in all test groups.

In 2013, Saheb-Al-Zamani et al. [49] investigated the use of a detergent based processed nerve allograft in a rodent animal using the transgenic Thy1-GFP Rat. Induced nerve defects for subsequent repair included a defect length between 2.0, 4.0, and 6.0 cm. Detergent based decellularization was performed with Triton X-200, sulfobetaine-16, and sulfobetaine-10. Autologous nerve grafting served as control group. The results demonstrated that axonal regeneration deteriorated with an increasing graft length. Saheb-Al-Zamani et al. [49] revealed that this is associated with an increasing expression of senescence markers within the autologous Schwann cells. The major results of this research project were the assumption that an increasing expression of senescence markers limit the axonal regeneration possibilities on critical nerve defects.

In accordance with the results of Saheb-Al-Zamani et al. [49] in 2013, the Poppler et al. [50] subsequently designed an experimental setting to explore the role of senescence cells during nerve regeneration on critical nerve defects (6.0 cm) in the rodent model. In this study, the expression of senescence markers was analyzed in long (6.0 cm), and short (3.0 cm) detergent based processed nerve allografts implanted in the transgenic Thy1-GFP rat. Used allografts were decellularized according to the protocols of Hudson et al. [33]. The results displayed that, due to the senescence of present Schwann Cells, a growth arrest occurred usually four weeks following surgery. In 2016, Poppler et al. [50] were able to demonstrate the senescence process within the allograft by replacing a 4.0 cm distal portion of the graft with a 4.0 cm autograft prior to the previously observed time point of growth arrest. No growth arrest appeared in these cases. As deduced from this approach, the senescence of invading Schwann cells and stroma cells and the resulting microenvironment in acellular nerve allografts might be the reason for a reduced outcome after the reconstruction of longer nerve defects.

Based on the decellularization process used by Poppler et al. in 2013 [50], the research group around Yan et al. [51] investigated the use of a 6.0 cm chemical processed nerve allograft compared to a hybrid-allograft constructed from an isograft and a chemical processed nerve allograft in the sciatic rodent model using Lewis rats [51]. Nerve regeneration was controlled after 4 and 20 weeks by evaluation of motor nerve recovery and histological analysis. Both groups resulted in poor outcome. Only moderate axonal regeneration was evaluable in the hybrid-allograft. Based on the data of Yan et al., 2018 [51], nerve regeneration using hybrid-allografts or chemical processed nerve allografts is limited due to graft length. The authors stated that only the autologous nerve graft facilitate axonal regenerated. 

### 3.3. Clinical Application of Allografts

In a single case study from 1996, Mackinnon described a nerve allograft reconstruction of a 9.0 cm tibial nerve defect after a lawn mower accident in a 12 year old male [52]. The patient received multiple cold-preserved allografts (20.0 cm cable graft) for reconstruction of the tibial nerve defect. A temporary immunosuppression for 14 months was performed using cyclosporine A and prednisolone. The follow up of the case demonstrated a sensible nerve recovery (9/10 on an individual scale) supported by a light motor function recovery at two years following surgery. Mackinnon et al. demonstrated a nerve regeneration in long distance nerve defects using cold preserved matched donor nerve allografts in combination with a temporary immunosuppression in this single case.

A subsequent case series from the same group in 2000, reported seven cases where peripheral nerve defects were reconstructed with cadaveric nerve allograft [53]. All cadaveric nerve allografts were cold preserved at 5 °C for 7 days in University of Wisconsin solution prior to transplantation. All hosts were immunosuppressed using tacrolimus, or cyclosporine A prior and afterwards to the reconstructive nerve surgery. Immunosuppression was maintained for 12–26 months following surgery. Where possible, transplanted nerves were ABO blood type matched. The repaired nerve defects were between 15.0–37.0 cm. Following surgery, three individuals regained light motoric function in the area of distribution. In 5 patients, regeneration was seen as the recovery of sensitivity that included vibration and light touch. One host rejected the allograft. Despite this achievement in allograft nerve reconstruction, Mackinnon et al., point out that the direct end-to-end suture or the autograft transplantation have to be preferred in a standard clinical setting.

### 3.4. Clinical Application of FDA Approved Allograft

To date, the Axogen Avance^®^ (AxoGen, Inc., Alachua, FL, USA) nerve allograft is the only FDA approved cadaveric decellularized nerve conduit for clinical application [11]. The “Registry of Avance^®^ Nerve Graft’s Utilization and Recovery Outcomes Post Peripheral Nerve Reconstruction” (RANGER) is the registration study of the Axogen Avance^®^ nerve graft [54]. The RANGER-study is registered under NCT01526681. The study started in 11/2008 and is planned to be finalized in 12/2025. Including criteria are nerve defects up to 7.0 cm in length reconstructed with Axogen Avance^®^ nerve graft.

Brooks et al., 2012 presented results of the RANGER study in a comprehensive clinical case report [55]. In this study, nerve defects reconstructed between 0.5–5.0 cm with Axogen Avance^®^ nerve graft were included. A meaningful recovery regarding motoric (≥M3) and sensitive function (≥S3) on MRCC scale [56] was observed in 87% of trial participants. Mean follow up was 264 ± 152 days. No allograft rejection was observed within the observation time.

In 2012, Cho et al. [57] reported results after bridging a mean gap length of 2.3 ± 1.2 cm (range 0.5–5.0 cm) with Axogen Avance^®^ nerve allograft in 51 patients. An improvement of sensory and motor function was detected in 86% of cases by the definition of a meaningful recovery. The predominant location was in digital area, whereas 15 cases of mixed, or motor nerves were affected. Noteworthy, MRCC scores of S3/M3 or better were gained in short (0.5–1.4 cm) and long (3.0–5.0 cm) gaps in 100%, and 90% of cases, respectively.

Zuniga et al., 2015 [58] analyzed the outcome of the Axogen Avance^®^ allograft in oral and maxillofacial nerve reconstruction. A total of 21 patients with lesions of lingual, and inferior alveolar nerve were included in this study. The diameters of applied grafts were in the range of 0.2 to 0.5 cm and the length averaged 3.42 ± 2.55 cm (0.8–7.0 cm). A meaningful recovery corresponding to a score of moderate, or better in the neurosensory test [59] was observed in 87% of all cases upon a follow up period of at least 6 months. In detail, short (0.8–2.0 cm) and long defects (3.0 to 7.0 cm) showed a similar outcome to (86% vs. 89%).

Salomon et al., 2016 [60] reported a case series including 7 patients that received an inferior alveolar nerve reconstruction of 5.0–7.0 cm using the Axogen Avance^®^ nerve allograft. Mean follow up time in this case series was 17.7 months. Patients that received a 7.0 cm nerve allograft underwent their follow up examination 10–24 month following surgery. 85.7% regained tactile sensation regarding to level S3. One patient (5.0 cm nerve reconstruction) regained S3+ sensory recovery (follow up = 27.5 months). In conclusion, the use of Axogen Avance^®^ nerve graft resulted in meaningful recovery after reconstruction of inferior alveolar nerve defects between 5.0–7.0 cm.

Rinker et al., 2017 [61] analyzed the results of the RANGER study with regard to nerve defects from 2.5–5.0 cm in the hand reconstructed with the Axogen Avance^®^ allograft. Successful nerve regeneration was seen in 86% of cases equivalent to a S3 level sensitive regeneration. Basically, this is in accordance with the achievements of the aforementioned studies.

Isaacs et al. [62], analyzed the RANGER data focusing on the outcome of nerve regeneration after nerve reconstruction using large diameter processed nerve allograft (Ø 4–5 mm). The reconstructed mean gap size was 3.3 ± 1.0 cm. The results indicate a meaningful recovery regarding motoric and sensory function independent to the allograft diameter in 67% or 85% of cases, respectively. Nevertheless, the authors concluded that this might be biased by a small population of 15 subjects.

Carlson et al., 2018 [63] reported single center results regarding the use of Axogen Avance^®^ nerve grafts on human nerve defects with an average defect length of 6.5 cm. Nerves were averagely reconstructed 12 weeks following trauma. 73% of the injuries were located in the upper extremity. The results indicate good to excellent regeneration regarding recovery of sensitivity in 91.7%. Mean follow up time was 15 month (±5). This is comparable to the clinical data reported by Rinker et al., 2017 [61], Brooks et al., 2012 [55], Salomon et al., 2016 [60], and Cho et al., 2012 [57]. Motoric nerve regeneration was graded as good in 33%. At this point, it has to be mentioned that only two clinical cases with a motoric nerve defect were included. However, the data presented by Carlson et al., 2018 [63] indicate that the use of allografts on critical nerve defect (6.5 ± 4.5 cm) might mediate sensible nerve regeneration successfully.

In 2020 Safa et al. [64], and Leckenby et al. [65] released the latest data from the RANGER study. Safa et al., 2020 [64] analyzed 624 nerve repairs in 385 subjects. Nerve gap length variated by 1.5–7.0 cm. 82% gained a meaningful recovery regarding motor and/or sensible function. The major conclusion based on the analyzed data was that nerve regeneration in the upper extremity lead to significant better results than in the lower extremity. The authors stated that a meaningful recovery can be achieved on nerve defects up to 7.0 cm in length.

Leckenby et al., 2020 [65] conducted a retrospective analysis of 129 patients with 171 implanted Axogen Avance^®^ nerve graft with a mean follow up time of 13 months (6 to 49 months). A meaningful sensory (≥S3), and motor recovery (≥M3) was achieved in 73.7%, and 40.1% of all implanted grafts, respectively. A significant discrepancy was seen between allografts implanted in under six weeks after nerve injury (≥M3 in 36% of cases) and two to six months (≥M3 in 100% of cases). Further, Leckenby et al. described a significant correlation between an inferior prognosis and a larger diameter as well as longer length. In one case of their study, a necrosis in the center part of the Axogen Avance^®^ nerve graft was detected on an explanted 4 × 50 mm^2^ graft. Consequently, the authors deduced a recommendation for using multiple smaller diameter grafts in preference to one larger diameter graft to prevent an insufficient perfusion.

**Table 2:** Clinical use of cadaveric allografts.

Length	Defect	Observation Time	Grouping	Immunosuppression	Cofactor/Cells	Outcome	Decellularization	Author
9.0 cm	tibial	diverse	allograft	Cyclosporine A	no	Sensitive regeneration after allotransplantation of tibial nerve	fresh cold preserved	Mackinnon 1996 [52]
12–37 cm	diverse	diverse	immuosuppressed allograft	Cyclosporine A, tacrolimus	no	Rejection in one Patient, sensory and motor recovery is possible	AB0 compatible, cold preserved at 5 °C in UW-Solution for 5 days	Mackinnon 2001 [53]
0.5–5.0 cm	diverse	diverse	allograft w/o control	no	no	Inferior to autograft, superior to artificial grafts	Axogen Avance^®^	Brooks 2011 [55]
0.5–5.0 cm	diverse	diverse	allograft	no	no	S3 and M3 or better was achieved in 86% of cases	Axogen Avance^®^	Cho 2012 [57]
0.8–7.0 cm Ø 2–5 mm	lingual and inferior alveolar nerve	diverse	allograft	no	no	87% had a sensory improvement in neurosensory test	Axogen Avance^®^	Zuniga 2015 [58]
5.0–7.0 cm	inf. alveolar nerve	17 month mean follow up time	allograft w/o control	no	no	S3-S3+ neurosensitive recovery. Best results in 5.0 cm	Axogen Avance^®^	Salomon 2016 [60]
2.5–5.0 cm	diverse	diverse	allograft	no	no	S3 or greater level was reported in 86% of repairs	Axogen Avance^®^	Rinker 2017 [61]
3.3 ± 1.0 cm Ø 4–5 mm	diverse	diverse	allograft	no	no	Successful regeneration on large diameter nerve defect (4–5 mm)	Axogen Avance^®^	Isaac 2016 [62]
Av. 6.5 cm	diverse	diverse	cadaveric decellularized allograft	no	no	Sensitive regeneration in 91.7%, motor recovery in 33%	Axogen Avance^®^	Carlson 2018 [63]
1.5–7.0 cm	diverse	diverse	allograft	no	no	Successful regeneration of motoric and sensitive nerve defect	Axogen Avance^®^	Safa 2020 [64]
0.8–10.0 cm Ø 1–5 mm	diverse	diverse	allograft	no	no	77% and 36% of patients showed S3, or M3 or better, respectively	Axogen Avance^®^	Leckenby 2020 [65]

## 4. Discussion

Early approaches in peripheral nerve reconstruction by unprocessed nerve allografts failed in different animal models [30,38]. Similar to a solid organ transplantation the use of unprocessed allografts lead to a significant graft rejection mediated by humoral or cellular immune reaction [14,66,67,68,69].

Over the time cold storage solutions evolved and the results after nerve allograft reconstruction improved. The widely used University of Wisconsin solution was able to enhance storage time and reduce immunogenic activity, but failed to support peripheral nerve regeneration in different contexts [40,48]. Especially the investigation of Strasberg et al. [40] in 1996 demonstrated that storage in University of Wisconsin (UW) solution alone is not able to enhance nerve regeneration after allograft transplantation on critical nerve defects. Nevertheless a downregulation of the expression of MHC II is observable [26].

A combination with a systemic immunosuppression was able to improve results in nerve regeneration but increased the risk of major side effects and opportunistic infections [43,45,47]. Nevertheless, a mild immunosuppression is discussed to enhance peripheral nerve regeneration [70]. Following the reviewed studies a combination of cold storage in UW-solution and mild immunosuppression is able to enhance and mediate nerve regeneration following allograft repair [45,47].

The detergent based decellularization is the current gold standard in allografting. The use of a decellularized allograft lead to significant neuronal regeneration in pre-clinical and clinical settings but is still inferior to the current gold standard, the autologous nerve transplantation [30,38,39,40,41,42,43,45,47,48,49,50]. In future experimental investigations it could be beneficial to combine a mild systemic or preferably even local immunosuppression with allograft transplantation which were decellularized by a detergent based process for peripheral nerve allografts.

In addition, it could be advantageous to overcome current limitation in length and diameter by adding autologous Schwann cells. Especially Saheb-al-Zamani et al., 2013 [49] and Brenner et al., 2005 [44] were able to demonstrate a positive effect on nerve regeneration by adding autologous or MHC-matched Schwann cells to cold stored nerve allografts. Recently, Kornfeld et al. [71] were able to demonstrate a delay in nerve regeneration by a time consuming repopulation of acellular nerve grafts by autologous Schwann cells compared to isograft control.

The use of human autologous Schwann cells for transplantation was first approved in the United Stated by the FDA for treatment of spinal cord injuries in 2012 [72]. A translation to the peripheral nervous system is ongoing. Promising results in the rodent model underline that the combination of autologous Schwann cells and artificial nerve grafts is beneficial for nerve regeneration in critical nerve defects [73]. Translation to the human is pending. A phase one study is currently recruiting participants for autologous nerve reconstruction in combination with an autologous Schwann cell transplantation (NCT03999424) [74].

Transferred to the legislation in Germany, the clinical translation of cell based methods is difficult to realize [75]. Basically, cell based transplantation is allowed by the law of the European Union but exceeded by the local German tissue act [75,76,77]. At the moment, processed autologous cell lines for clinical use have to be certified as a drug accordingly to the German “Arzneimittelgesetz” (German medicinal products act). Processing of drugs is only allowed in approved facilities following §13 of the German medicinal products act. As a result of this highly regulated and expensive process, in 2020 no procedure for autologous Schwann cell transplantation was registered for clinical use in the federal republic of Germany. However, the recent approval of autologous chondrocytes transplantation as a pharmaceutical drug for cartilage repair in knee injuries gives a positive outlook [78,79]. Future application of autologous Schwann cell transplantation is critically dependent on changes in national legislation. Nevertheless, the reviewed studies demonstrated that a combination of cold storage in UW-Solution and a combination of autologous Schwann cells is able to enhance axonal regeneration in absence of immunosuppression.

Furthermore, the urgent need for supporting factors or myelinating cells to overcome senescence of resident cells is underlined by Saheb-al-Zamani et al., 2013 [49] in the context of critical nerve defects. This is one of the main aspects to highlight Schwann cell transplantation into the focus of research. Recently published data of Santosa et al., 2013 [80] demonstrated that nerve regeneration through a 14 mm allograft, populated with glial cell line derived neurotrophic factor (GDNF), lead to overexpressing Schwann cells in the Lewis and Sprague–Dawley rat. Regeneration was controlled after 6 and 12 weeks. The results indicate a comparable regeneration in the allograft group compared to the current clinical gold standard. Furthermore, promising materials such as spider silk [81], and multipotent cells such as mesenchymal stem cells [82,83], or stem cell-derived extracellular vesicles [84,85,86] could further optimize the outcome following allograft nerve repair.

In clinical settings the use of cadaveric processed nerve graft or artificial nerve graft is beneficial for patients to avoid donor side morbidity or offers new reconstructive perspectives in cases where donor nerves are not available or limited [11,87,88]. Further, a minor, but important share of trauma patients suffers from multiple nerve lesions as well as patients with various types of neurofibromatosis [4,89,90]. This kind of patient would profit from a limitless reserve of donor nerves which is enabled by the use of artificial nerve grafts.

Clinical results following Axogen Avance^®^ engraftment are encouraging. Most reviewed studies were able to achieve a meaningful recovery in upper 8th percentile of all cases [55,57,58,60,62,63,64]. Satisfactory motor recovery was reported in 40.1% to 74%. Data for reconstruction of critical nerve defects extending 5.0 cm in length by Axogen Avance^®^ are scarce at the moment [58,60,65]. Following Carlson et al. [63] and Rinker et al. [61], the use of Axogen Avance^®^ to this point of research is recommended for defects of less than 5.0 cm in length [61,63].

At this point, the question has to be raised whether a detergent based decellularized/processed nerve allograft is superior to other marketed artificial nerve grafts such as Neurotube^®^ (Neuroregen L.C.C, Bel Air, MD, USA), NeuroGen^®^ (Integra Lifescience Corporation, Plainsboro, NJ, USA) or Reaxon^®^ (Medovent GmbH, Mainz, Germany). Whitlock et al., 2008 [36] was able to demonstrate that an FDA approved allograft did not show superior regeneration in direct comparison to NeuraGen^®^ (Integra Lifescience Corporation, Plainsboro, NJ, USA). After reconstruction of a 1.4 cm sciatic nerve defect in the Lewis rat no significant difference in axon count was verifiable following 12 weeks of surgery. The isograft still demonstrated superior regeneration compared to NeuraGen^®^ and Axogen Avance^®^. Thus, on a 2.8 cm sciatic nerve defect, regeneration after surgical reconstruction by the collagen-based nerve graft was inferior to the allograft group, and the isograft control. Basically this is in agreement with the previously reviewed pre-clinical studies [30,38,39,40,41,42,43,45,47,48,49,50]. Beside this, there is still a lack of evidence that processed allograft can perform superior to other marketed materials.

In summary, the autologous nerve transplantation still demonstrated superior results to the pre-clinical and clinical use of artificial nerve grafts and processed/unprocessed allografts. Detergent based processed allografts are considered to facilitate nerve regeneration and hamper implant rejection. Despite the known side effects, immunosuppression is associated with a better outcome for axonal regeneration.

## 5. Materials and Methods

Systematic Review was conducted in accordance with the PRISMA statements.

### 5.1. Including Criteria

Only scientific work in regard to peripheral nerve regeneration/reconstruction was included. The main criteria was a surgical nerve reconstruction in small/large animal models with cadaveric nerve allografts on nerve defect sizes ≥4.0 cm.

### 5.2. Matches

In all, 29 articles met the inclusion criteria of nerve reconstruction on nerve defects ≥4.0 cm (15 original articles and 11 clinical reports). Two records were excluded after identified as duplicates. One article was removed due to incomparable methods.

### 5.3. Literature Search

A literature search was performed via PubMed and Google Scholar. A key word search was performed using the following words: “nerve allograft”, “long segment nerve defect”, “long length nerve defect”, “critical nerve defect”.

## 6. Conclusions

Autologous nerve transplantation is the gold standard for reconstruction of critical nerve defects but limited in length and associated with donor side morbidity. Clinical data of processed nerve allografts indicate a solid, and reliable axonal regeneration on non-critical nerve defects. Furthermore, processed nerve allograft transplantations are at least equal but not superior to the autologous nerve transplantation on non-critical defect sides. At this point of research, the processed nerve allografts can be seen as an alternative in cases where isograft material is limited in quantity or to avoid donor side morbidity. An appraisal of whether processed nerve allografts perform superior to other marketed and FDA approved materials is not implied by the referenced literature. Based on the reviewed data, marketed allografts are a useful addition to the “of the shelf products”. Data on long length nerve defect reconstruction extending 5.0 cm by processed allografts is rare. Therefore, a reconstruction of critical peripheral nerve defects by allografts as an alternative to autologous nerve transplantation in clinical settings cannot be recommended, yet. More clinical data is needed. For future developments, the combination of acellular allografts/artificial nerve graft with autologous Schwann cells might be beneficial to optimize results.

## Figures and Tables

**Table 1:** Pre-clinical use of cadaveric allografts

Length	Defect	Animal	Observation Time	Grouping	Immunosuppression	Cofactor/Cells	Outcome	Decellularization	Author
1–4 cm	peoneal	rat	90 days	allograft vs. autograft	no	no	Full regeneration in immunosuppressed specimen	unprocessed	Zalewski et al., 1982 [30]
4 cm	tibial	rat	2–9 month	allograft vs. autograft	no	no	Only fresh autograft demonstrated sufficient regeneration	frozen, unprocessed	Zalewski et al., 1982 [38]
2 cm	peroneal	rat	1, 2, 4, 12, weeks	allograft vs. autograft	no	no	Regeneration of autograft is superior to acelluar allografts	freeze-thawing	Gulati et al., 1994 [39]
4 cm	peroneal	rabbit	1, 2, 4, 12, 20 weeks	allograft vs. autograft	no	no	Regeneration of autograft is superior to acelluar allografts	freeze-thawing	Gulati et al., 1994 [39]
8 cm	median	sheep	6 and 10 month	allograft vs. autograft	no	no	Superior regeneration in fresh autograft. Failed to regenerate in rest	unprocessed, cold preserved	Strasberg et al., 1996 [40]
8 cm	ulnar	porcine	6 and 10 month	allograft vs. autograft	no	no	Regeneration in autograft, rejection of allograft	unprocessed	Atchabahian et al., 1998 [41]
5 cm	peroneal	canine	1 and 3 month	allograft vs. autograft	no	bFGF	Freeze thawed acellular allograft results in peripheral nerve regeneration	freeze-thawing	Ide et al., 1998 [42]
8 cm	median	sheep	35–47 days	immunusuppressed allograft/autograft vs. allograft/autograft	Cyclosporine A	no	Truncation due to overwehelming side effects of immunosuppression	fresh unprocessed	Matsuyama et al., 2000 [43]
5 cm	ulnar	porcine	20 weeks	MHC matched SC in cold preserved allograft with UVB irradiated donor alloantigens	no	MHC matched SC	Robust nerve regeneration by combination of cold preservation + MHC SC transplantation, no superior regeneration by adding alloantigens UVB	cold preserved	Brenner et al., 2004 [44]
8 cm	ulnar	porcine	24 weeks	allograft vs. autograft	Tacrolimus (FK506)	no	Regeneration in FK05 immunosuppressed allograft, no regeneration in allograft control, 50% of immunosuppressed animals sacrificed prior to experimental endpoint	unprocessed	Jensen et al., 2005 [45]
6 cm	median	porcine	10 month	allograft vs. autograft	UV-B	no	Regeneration in pretreated groups, no regeneration wo immunosuppression	unprocessed	Tung et al., 2006 [46]
4 cm	ulnar	primate	8 month	allograft vs. autograft	Tacrolimus (FK506)	no	No statistical difference, lower NCV--> partial rejection of implant, nevertheless complete regeneration after 8 month	cold preserved	Auba et al., 2006 [47]
6 cm	ulnar	primate	6 month	fresh and cold preserved allograft vs. autograft, vs. SC transplanted cold preserved allograft	no	no	Successful regeneration in cold preserved allograft, superior regeneration in cold preserved allograft + SC, not superior to autograft	cold preserved	Hess et al., 2006 [48]
2.0, 4.0, 6.0 cm	sciatic	rat	10–20 weeks	allograft vs. autograft	no	no	SC senescence hampers regeneration in long decellularized allografts	chemical decellularization	Saheb al Zamani et al., 2013 [49]
3–6 cm	sciatic	rat	-	allograft vs. allograft + autograft	no	no	Growth arrest due to senescence in long nerve allograft	chemical decellularization	Poppler et al., 2016 [50]
6 cm	sciatic	rat	4 and 20 weeks	hybrid allograft vs. allograft	no	no	no superior regeneration in hybrid ana, autograft is still the method of choice	chemical decellularization	Yan et al., 2018 [51]

## Data Availability

The data presented are available within the article.

## Abbreviations

bFGF	fibroblast growth factor
EC-solution	Euro-Colins Solution
FK506	Tacrolimus
GDNF	glial cell line derived neurotrophic factor
NCV	nerve conductive velocity
MHC II	Major histocompatibility complex II
UW-solution	University of Wisconsin solution
